# A Non-Destructive Method for Distinguishing Reindeer Antler (*Rangifer tarandus*) from Red Deer Antler (*Cervus elaphus*) Using X-Ray Micro-Tomography Coupled with SVM Classifiers

**DOI:** 10.1371/journal.pone.0149658

**Published:** 2016-02-22

**Authors:** Alexandre Lefebvre, Gael Y. Rochefort, Frédéric Santos, Dominique Le Denmat, Benjamin Salmon, Jean-Marc Pétillon

**Affiliations:** 1 De la Préhistoire à l'Actuel, Culture, Environnement, Anthropologie—UMR 5199, University of Bordeaux, Bordeaux, France; 2 EA 2496 Pathologies, Imagerie et Biothérapies oro-faciales, Plateforme Imagerie du Vivant, Dental School, Université Paris Descartes Sorbonne Paris Cité, Montrouge, France; 3 Assistance Publique des Hôpitaux de Paris (AP-HP), Hôpitaux Universitaires PNVS, Service d’Odontologie Bretonneau, Paris, France; 4 Travaux et Recherches Archéologiques sur les Cultures, les Espaces et les Sociétés-UMR 5608, University Toulouse Jean Jaurès, Toulouse, France; Museo Nazionale Preistorico Etnografico 'L. Pigorini', ITALY

## Abstract

Over the last decade, biomedical 3D-imaging tools have gained widespread use in the analysis of prehistoric bone artefacts. While initial attempts to characterise the major categories used in osseous industry (i.e. bone, antler, and dentine/ivory) have been successful, the taxonomic determination of prehistoric artefacts remains to be investigated. The distinction between reindeer and red deer antler can be challenging, particularly in cases of anthropic and/or taphonomic modifications. In addition to the range of destructive physicochemical identification methods available (mass spectrometry, isotopic ratio, and DNA analysis), X-ray micro-tomography (micro-CT) provides convincing non-destructive 3D images and analyses. This paper presents the experimental protocol (sample scans, image processing, and statistical analysis) we have developed in order to identify modern and archaeological antler collections (from Isturitz, France). This original method is based on bone microstructure analysis combined with advanced statistical support vector machine (SVM) classifiers. A combination of six microarchitecture biomarkers (bone volume fraction, trabecular number, trabecular separation, trabecular thickness, trabecular bone pattern factor, and structure model index) were screened using micro-CT in order to characterise internal alveolar structure. Overall, reindeer alveoli presented a tighter mesh than red deer alveoli, and statistical analysis allowed us to distinguish archaeological antler by species with an accuracy of 96%, regardless of anatomical location on the antler. In conclusion, micro-CT combined with SVM classifiers proves to be a promising additional non-destructive method for antler identification, suitable for archaeological artefacts whose degree of human modification and cultural heritage or scientific value has previously made it impossible (tools, ornaments, etc.).

## Introduction

Studies on the characteristics of prehistoric osseous material contribute to a better understanding of prehistoric societies and help to reconstruct the economic, technical, and symbolic interactions between human groups and the animal world [1–4]. Among the osseous material available in the surrounding environment (i.e. bone, antler, and dentine/ivory), antler was used throughout the Upper Palaeolithic in Western Europe (40,000–11,000 years cal BP) in the manufacture of a significant proportion of hunter-gatherer equipment. As antler withstands the constraints of flexion better than other osseous material, it was particularly prized for the production of projectile points, due to its higher impact resistance [5–14].

While it is now possible to distinguish between the major categories used in bone industry [15–17], the taxonomic determination of prehistoric remains is still to be investigated. For instance, regarding antler artefacts, the distinction between reindeer (*Rangifer tarandus*) and red deer (*Cervus elaphus*) antler, which are the two main species whose antlers were exploited, can be challenging when the material is fragmented or has been modified by human activity. However, the identification of these raw materials could help to indicate the acquisition strategies of particular groups, specific technical know-how, and even symbolic choices in relation to species, where both species were available in the environment.

In terms of cross-border relations between prehistoric Magdalenian groups (between 19,000 and 14,000 years cal BP) on either side of the Pyrenees (France/Spain) for example, the question of the comparative use of antler is of particular interest in our understanding of the technical and economic interactions between prehistoric groups in relation to environmental variations on either side of a natural ecological and climatic barrier [18–23]. During the Magdalenian, the distribution of deer was affected by the Pyrenees mountain range: reindeer were abundant in the north, but much less common to the south of it, while red deer largely dominated the faunal spectra in the south but were less present in the north. Magdalenian hunter-gatherers on both sides of the Pyrenees were in close interaction however. In such a situation, the identification of raw materials could indicate the specific adaptation strategies of human groups and the possible circulation of antler throughout the Pyrenees.

### The value of X-ray micro-tomography in archaeological studies

Over the past decade, emerging technologies have helped to improve our understanding of the structure of prehistoric osseous material with microscopy [24, 25] and synchrotron imaging [16, 26–28], and that of their chemical composition with isotope ratios [29], DNA analysis [30], micro PIXE/PIGE [15], and mass spectrometry [31–35]. Considering the "sensitive" nature of some archaeological artefacts (due to their fragility, rarity, heritage value, or scientific importance), X-ray micro-tomography offers new perspectives for analysis. Derived from 3D biomedical imaging, micro-Computed Tomography (micro-CT) allows for the production of three-dimensional high-resolution, images and measurements of the internal structures of material in a non-invasive and non-destructive manner [36–38]. Nowadays, its main applications in prehistoric archaeology are in the fields of physical anthropology [39–47], the identification of taxonomic markers [16, 17, 48] and the recognition of technical or functional traces [49–51].

### Criteria established for the characterisation of antlers

#### External macroscopic criteria

**Fig 1:**
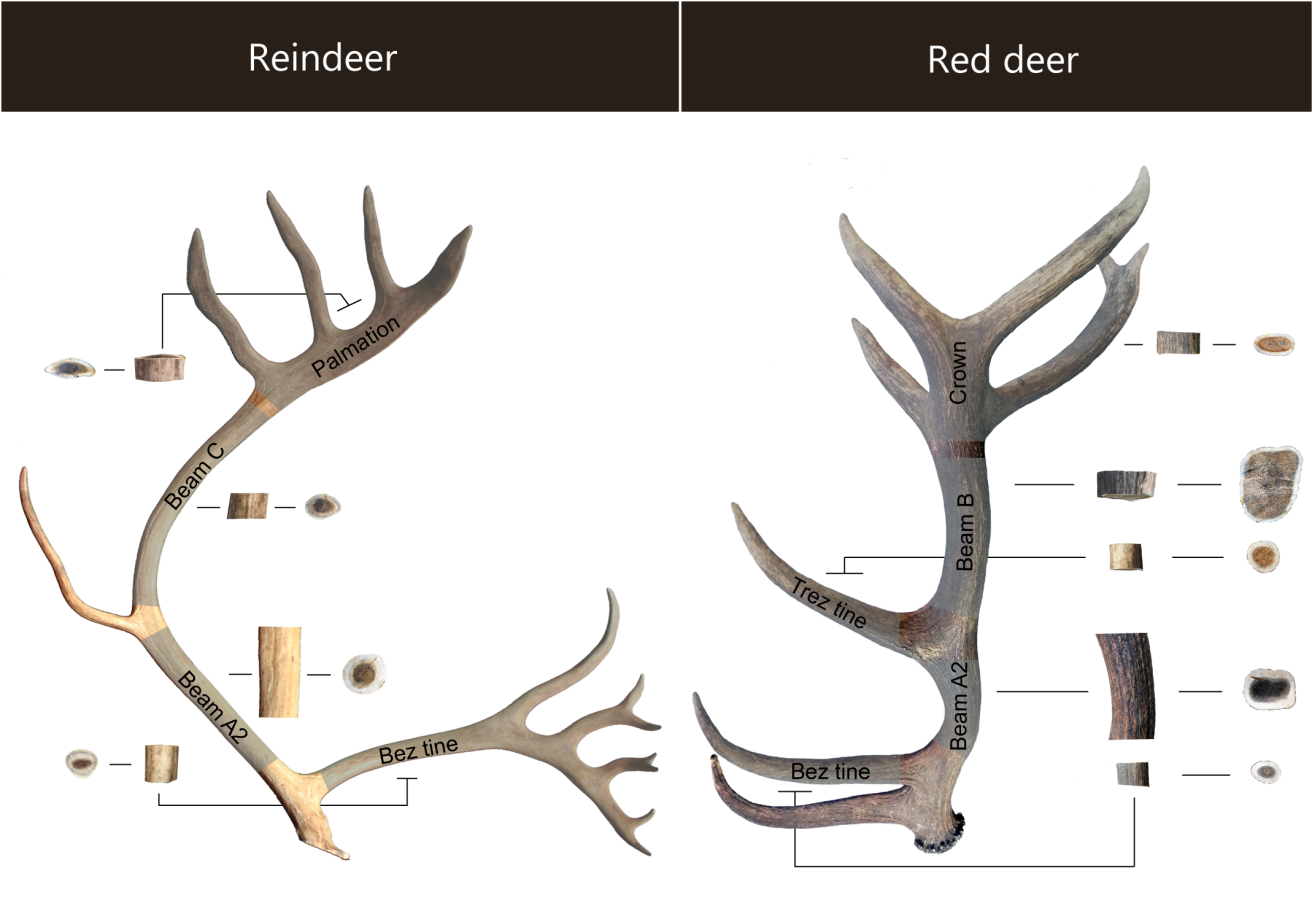
Anatomical location of the antler samples (modern corpus).

The distinction between reindeer and red deer antler is mainly based on the observation of macroscopic anatomical criteria. Firstly, the antlers differ significantly in their general morphology (sections, curvatures, etc.) (Fig 1). The ratio between the thickness of the compact tissue and the spongy tissue is particularly relevant here, and for the same anatomical position on the antler, this ratio is generally higher for reindeer [52, 53]. Overall, the most distinguishing element is the presence of pearling on the surface of red deer antler, corresponding to the remains of the external vascularisation system from the velvet present during the first months of antler growth [53]. This gives red deer antler its rough appearance, and is absent on reindeer antler.

Distinguishing directly between the antlers of both species becomes more challenging when the material is fragmented or has been modified by human activity (i.e. antler industry) or by post-depositional taphonomic processes associated with the long-term burial of remains.

#### Internal microscopic criteria

Two sets of distinguishing criteria have been proposed at a microscopic level. According to Penniman [52], the compact tissue of red deer antler presents a "more granular and less dense structure in the longitudinal section" than reindeer antler, while Averbouh [53] has established histological differences in terms of the osseous tissue: the grain of compact tissue being rougher for red deer and the mesh of trabecular tissue appearing tighter for reindeer. However, these criteria are unreliable without quantified biometric data.

To overcome the lack of non-destructive methods, we carried out a comparative biometric analysis to establish the relevant criteria for differentiating between red deer and reindeer antler. For this, we used trabecular bone microstructure analysis derived from biomedical micro-CT applications. The method was first validated using known modern samples and then assessed using our identified archaeological corpus.

## Materials and Method

### Samples

#### Modern corpus

In order to better define the anatomical and histological variability of antler, we compiled a complete biometric reference collection. Thirty-nine samples of modern antler (17 red deer antler and 22 reindeer antler) were collected from 28 individuals (S1 Table) of different weights and ages, ranging from one to six years in age. Semi-domesticated red deer antler was obtained from a farm in the Charentes region (southwest of France) and wild specimens from the forests of the Dordogne (southwest of France). Three farms, one in Finland (Ostrobothnia) and two in France (Orne, Alpes-de-Haute-Provence), provided the semi-domesticated reindeer antlers (more information on geographical location of the farms is available in S2 Table).

This collection does not require any ethic committee agreement insofar as it does not involve living animals: no animals were sacrificed or mistreated and no endangered species were involved. Moreover, the retrieval of the samples was performed in accordance with the relevant European legislation. In accordance with Article 547 of the French Civil Code relative to the gathering/collection of antlers, we obtained authorisation from the various private landowners to collect shed antlers within the French territory. As regards the recovery of Finnish reindeer antlers, their acquisition was made via the website "brisa.fi" in accordance with the Finnish legislation. Finally, red deer antlers from slaughtered animals were given to us by the association "Société de chasse de Lasserre" (24290, St-Amand-de-Coly, Dordogne, France) included in the National Hunting and Wildlife Office according to the French legislation on hunting.

Most of the samples were machine turned from the core of the antler's main beam (A2) located between the bez and trez tine in red deer and between the bez and posterior tine in reindeer (Fig 1). Anatomical variability was taken into account by including tine and crown/palmation elements (N = 6) and by scanning adjacent locations on identical samples with a known origin (S1 Table, in blue). Overall, 33 fully mineralised fragments were analysed, 12 of which came from shed antlers and 12 from the antlers of slaughtered animals (9 were unidentified). Once shaped, the cylinders were washed with high-pressure water and then cleaned with hydrogen peroxide.

It should be noted that three samples were dismissed from the study at the time of 3D acquisition due to the presence of visible fissures in the material (cf. 4. *Conservation may impact classification)*.

#### Archaeological corpus

**Fig 2:**
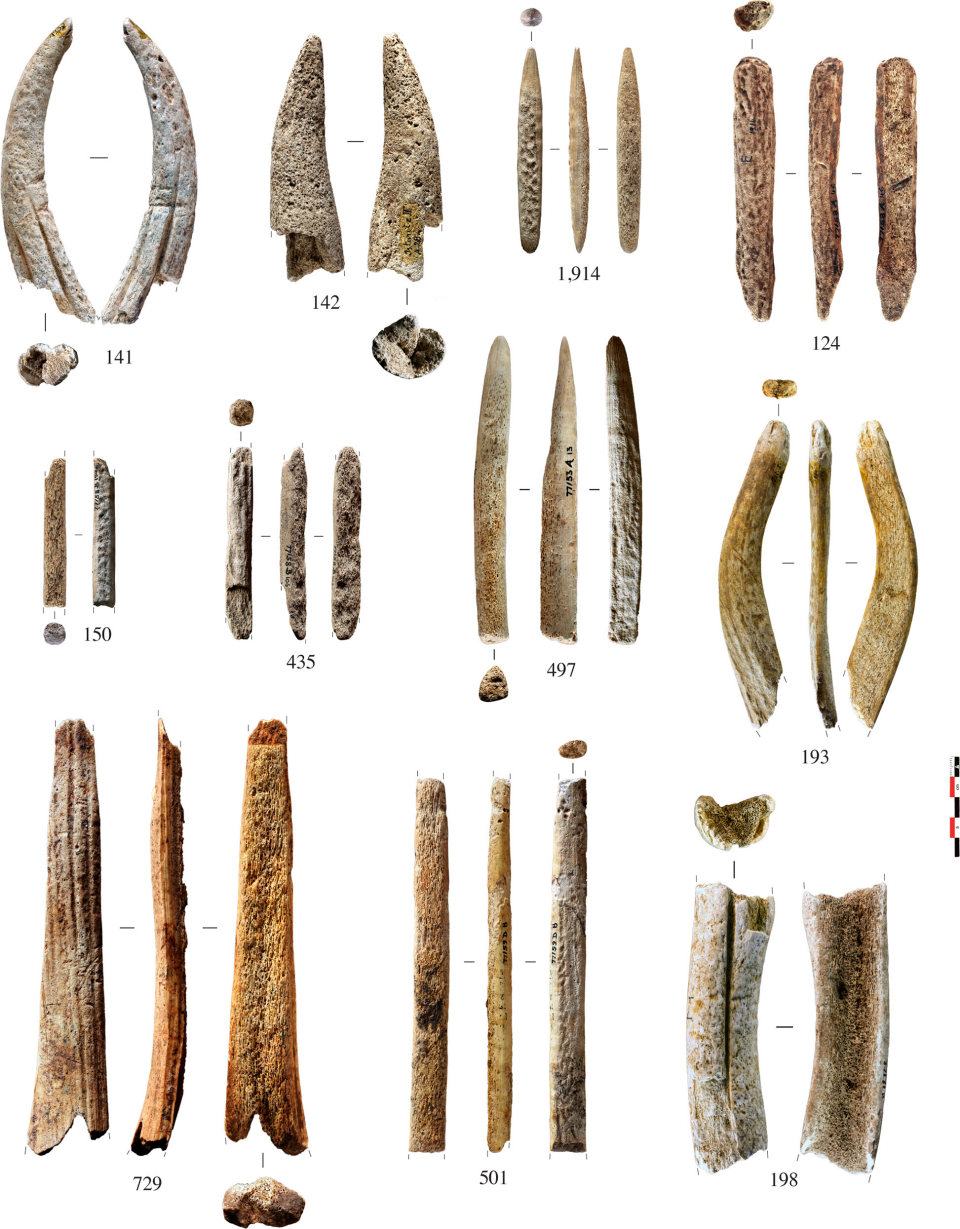
Red deer antler artefacts from the Magdalenian layers (E, II, I, F1) of Isturitz cave sampled for this study. Projectile points: 1,914. Half-round rod from tine element: 193. Unidentified rod fragments: 150, 435 and 501. Wedges from rod: 124 and 497. Flat blank: 729. Manufacturing wastes from tine (141) and beam (198) elements. Tine tip element: 132.

**Fig 3:**
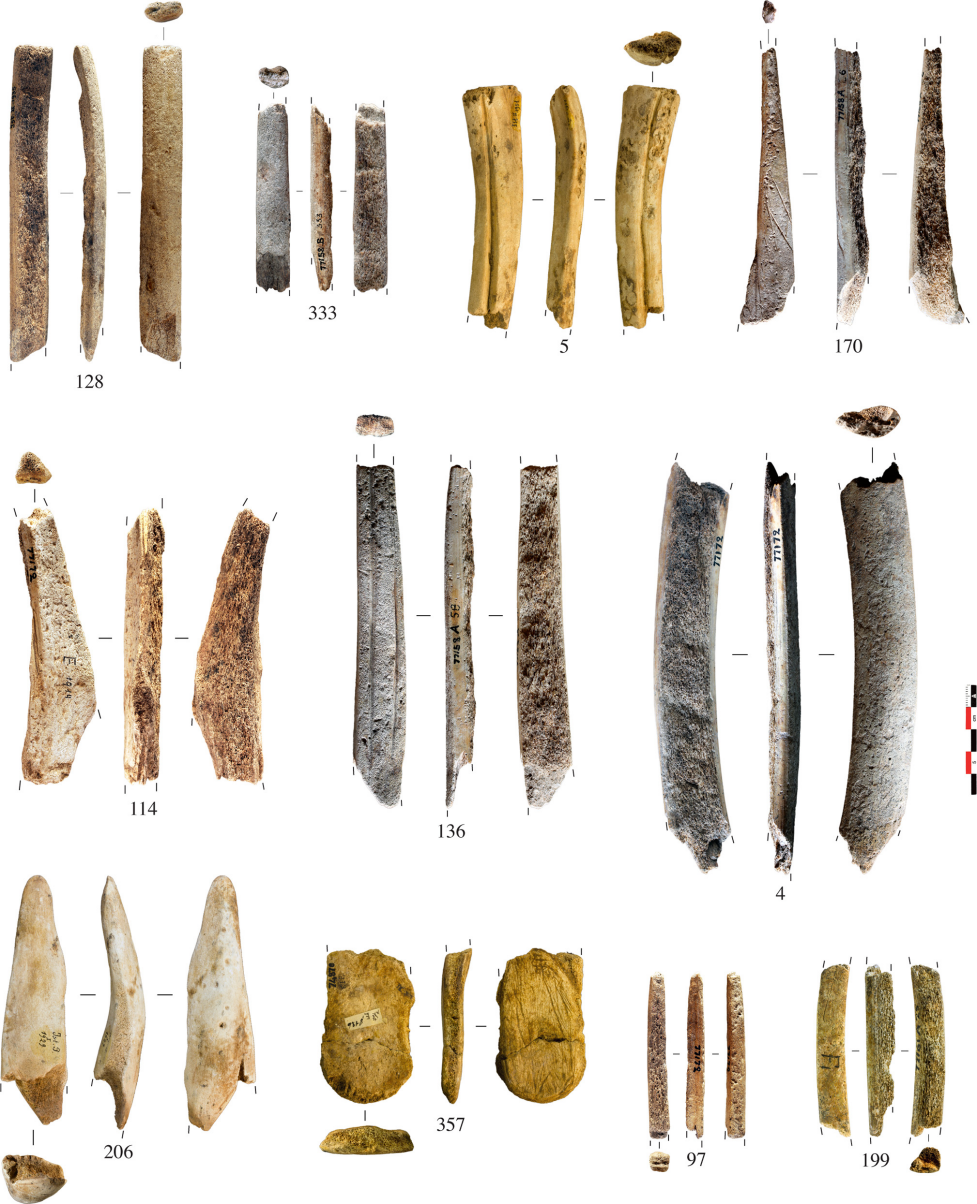
Reindeer antler artefacts from the Magdalenian layers (E, II, I, F1) of Isturitz cave sampled for this study. Projectile points: 97. Unidentified rod fragments: 170, 114, 333, 136, and 199. Flat blanks: 128 and 4. Manufacturing wastes from tine (5), tine tip (206), and palmation (357) elements.

Fifty antler artefacts were collected from the middle/upper Magdalenian layers (E, II, I, and F1) of Isturitz cave (Pyrénées-Atlantiques, France) (S3 Table). Excavated in the first third of the 20^th^ century, this archaeological material is now curated in the “Musée d’Archéologie Nationale” (MAN, Yvelines, France). The taxonomic origin of the artefacts was ascertained using the macroscopic criteria described above (cf. 1.2. Criteria established for the characterization of antlers). All of them present at least one unworked surface from which we can establish an obvious taxonomic diagnosis with the presence (or lack) of pearling [53]: 23 were from red deer antler (Fig 2) and 27 from reindeer antler (Fig 3). Most of them were rod-type products of debitage. Within the sample, 18 came from the beam, 12 from the peripheral anatomical portions (tine, tine tip, crown/palmation), and 20 were anatomically unidentified. The samples were all washed with distilled water and under sonication to remove sediment.

It should be noted that one archaeological sample was discarded during the 3D analysis due to sediment within the trabecular tissue that was impossible to remove despite sonication (cf. 4. *Conservation may impact classification)*.

### Micro-CT dataset acquisition

X-ray micro-tomography is a non-invasive imaging technique that explores the internal structure of (bio)materials based on radiodensity properties. Samples were scanned using a high-resolution X-ray micro-CT device (Quantum FX Caliper, Life Sciences, Perkin Elmer, Waltham, MA, United States). Three-dimensional acquisitions were performed using an isotropic voxel size of 20x20x20 μm^3^ (90 kV, 160 microA, 180 s).

### Image processing

For each sample, the micro-CT volumetric acquisition provided a stack of 512 cross sections. The multiplanar reconstruction tools allowed grey-level images to be displayed with an axial orientation. The lowest grey/dark pixels correspond to empty spaces and the highest grey/bright pixels to the densest/mineralised tissues.

#### Region of interest (ROI)

**Fig 4:**
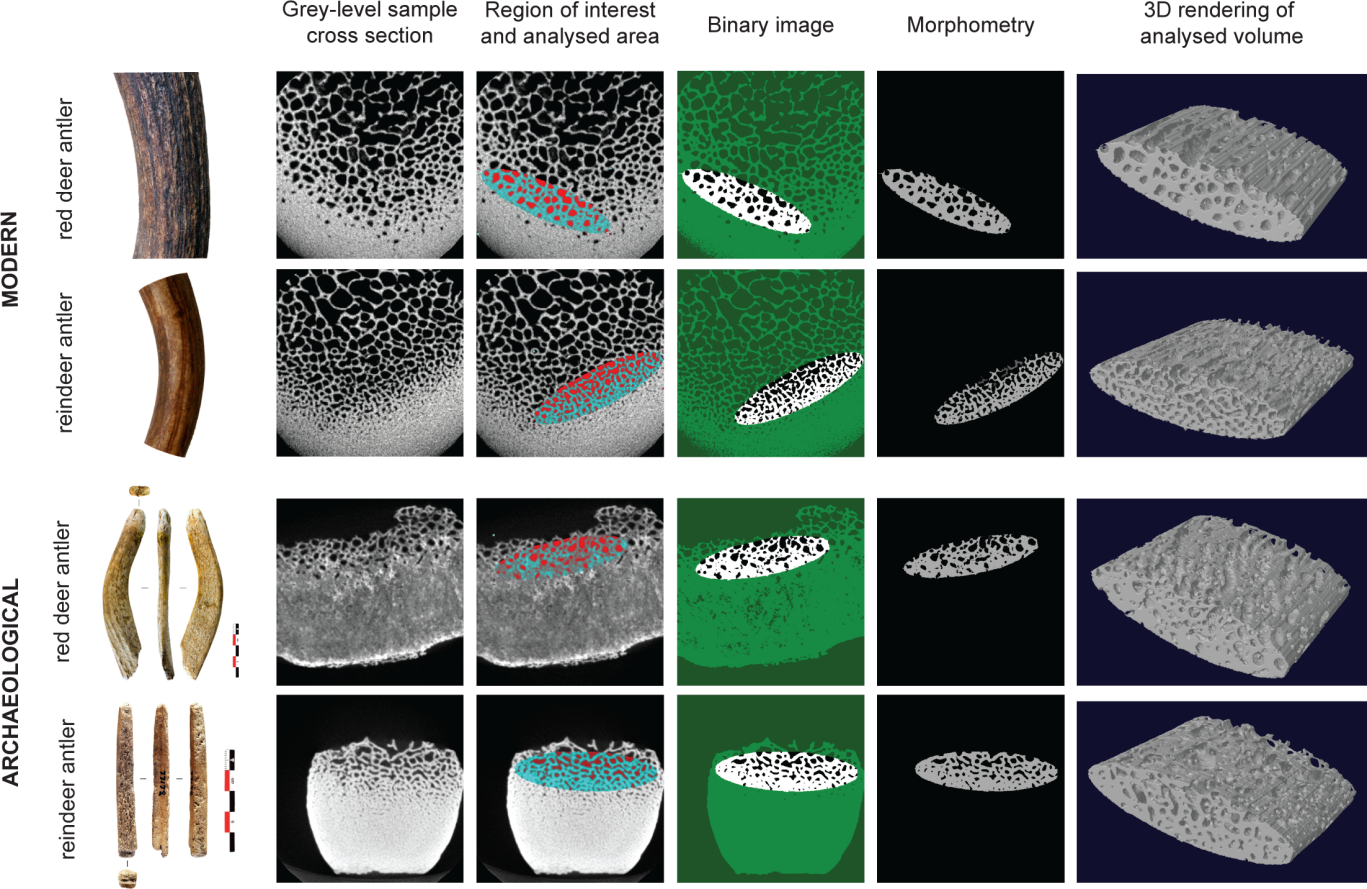
Image processing steps including the selection of the volume of interest (VOI) and the segmentation principle. The 3D acquisitions were performed with an isotropic voxel size of 20x20x20 μm^3^ (90 kV, 160 microA, 180 s) using a high-resolution X-ray micro-CT device. The same elliptical VOI (132.9 mm^3^), still positioned at the same location (at the junction between the central trabecular tissue and the external compact tissue), was applied to all the 39 modern samples and 50 archaeological ones (both corpuses are presented in S1 and S3 Tables). The same global binary threshold was applied to each data stack in order to isolate the mineralised and non-mineralised structures for subsequent quantitative biometric analysis including BV/TV, Tb.N, Tb.Th, Tb.Sp, SMI and Tb.Pf.

The analysed area is located at the junction between the central trabecular tissue and the external compact tissue (Fig 4). This histological area, defined as a "transition zone" by Rolf and Enderle (1999) [54], looks similar to trabecular bone-like tissue. For the archaeological corpus, this area corresponded to the residual part of the trabecular tissue present on the lower surface of the objects made on flat blanks (Fig 4).

This juxta-cortical area was cropped with a fixed ellipse of 12.98 mm^2^ (the radius of the minor axis = 1.07 mm and the radius of the major axis = 3.86 mm) in each 2D section of the stack with the dedicated shape tool in the “CT analyzer” software (Skyscan, release 1.13.5.1, Kontich, Belgium). This elliptical ROI was consistently placed tangentially to the most external visible alveoli organised in a homogeneous network, so that the whole surface of the shape was uniformly filled with alveoli (Fig 4). The 3D interpolated ellipses defined the volume of interest (VOI, 12.98 x 512 x 0.02 = 132.9 mm^3^ –ROI surface x number of slices x thickness of slice), which included the trabecular bone close to the peripheral cortical bone.

#### Segmentation

The same global binary threshold was applied to each data stack in order to isolate the mineralised and non-mineralised structures for subsequent quantitative analysis (Fig 4). This standardised threshold was automatically calculated from the arithmetic mean between the bone peak (white pixels) and the empty space peak (black pixels) of the VOI histogram.

### Morphometric assessment

The trabecular bone morphometric indices were extrapolated to investigate the micro-architecture of the antler samples. Calculation of the 3D structural parameters followed the guidelines of the American Society for Bone and Mineral Research [55] and were derived from the standardised nomenclature for bone histomorphometry originally described by Parfitt et al. (1987) [56] and recently updated by Dempster et al. (2013) [57]. Morphometric measurements were performed using the “CT analyzer” software (Skyscan, release 1.13.5.1, Kontich, Belgium) including:

- Bone volume fraction or bone volume/tissue volume ratio (BV/TV, %) to estimate the percentage of segmented mineralised volume to the total volume of interest, i.e. the white to black voxel ratio of the binary images.
- Trabecular number (Tb.N, mm^-1^) to measure the average number of mineralised structures per unit length.
- Trabecular thickness (Tb.Th, mm) to measure the mean thickness of the mineralised structures.
- Trabecular separation (Tb.Sp, mm) to calculate the average distance between mineralised structures, i.e. background (empty space) mean thickness.
- Structure model index (SMI) to gain information on the predominant shape (plate- versus rod-like) of the trabecular bone; the SMI was designed to be 0 for perfect plates, 3 for perfect rods, and 4 for ideal spheres [58]. The SMI was negative in the case of concave surfaces.
- Trabecular bone pattern factor (Tb.Pf, mm^-1^) [59] (or connectivity [60]) to establish the redundancy of mineralised structure connections, based on the relative ratio between the concavity and convexity of the surface.

### Statistical analysis

#### Support vector machines

Support vector machines (SVM) are modern non-probabilistic classifiers. The principle of standard SVMs is to identify a hyperplane for the optimal linear division of two groups of individuals. This hyperplane is constructed to maximise the distance, known as margins, between the nearest individuals of each group. Vapnik-Chrvonenkis theory [61] ensures that the larger the margins, the lower the rate of classification error.

In practice, there is little chance of the two groups being linearly separable in the original space where the individuals are defined by the initial variables. So the data must be reclassified in a higher dimensional space (*p*-dimensional) using a nonlinear function *φ*, which includes a linear separation. Since a perfect linear separation is rarely established, a variant of the method, called soft margin SVM, is usually applied. This variant allows for some classification error in the feature space [62].

The separating hyperplane can be defined as *w*^T^ *φ(x) + w*_*0*_ *=* 0, in which *w* and *w*_*0*_ are *p*-dimensional vectors respectively representing weight and intercept, and *x* is a *p*-dimensional vector providing the coordinates of an individual in the original space. This hyperplane provides a discrimination rule based on the associated function *h(x) = w*^T^ *φ(x) + w*_*0*_. If *h(x) >* 0, then the individual belongs to the first group, while if *h(x) <* 0, the individual belongs to the second group.

SVM classification requires us to choose a specific kernel function in order to calculate the scalar products *φ(x*_*i*_*)*^T^.*φ(x*_*j*_*)* and define the hyperplane. Here, we selected the Gaussian kernel, which has two parameters: *c* (tolerance to classification errors in the feature space) and *γ* (variance parameter for the Gaussian function). The optimal values for these two parameters must be identified in order to obtain the best classification, as the hyperplane directly depends upon them. The usual way is to define a grid search composed of powers of both parameters and to retain the pair leading to the best accuracy among all the possible values of the grid.

More information on SVMs can be found in Izenman (2008) [63].

#### Study design

After determining how individuals are distributed for each biometric variable (normality test), inter-group comparisons were calculated (Wilcoxon test) to highlight their “isolated” discriminative value (Table 1; S1 and S2 Figs). A Principal Component Analysis (PCA) was then performed to explore the correlations between all the variables and the scattering of individuals within and between species. Following this, an initial SVM model was built using the modern corpus. To assess the predictive performance of the model, we used leave-one-out cross-validation (LOOCV) to establish a classification error rate. We then applied the classification rule established from the modern corpus to the archaeological corpus to determine whether the characteristics distinguishing the two species were invariant over time. Finally, a second SVM model was created using only the archaeological corpus. All the biometric variables (BV/TV, Tb.N, Tb.Sp, Tb.Th, Tb.Pf, and SMI) were systematically used for the model: unlike with logistic regression, variable selection is not required for SVM.

**Table 1:** Mean and standard deviation of the biometric measurements and inter-group comparisons (Wilcoxon test) for the modern corpus. It included 39 antler samples of which 17 were red deer samples and 22 were reindeer samples (S1 Table).

			Reindeer		Red deer		Wilcoxon test
Biometrical parameters			*Mean*	*SD*	*Mean*	*SD*	*P-values*
Trabecular number	*Tb*.*N*	*1/mm*	3.51	0.37	2.83	0.45	0
Trabecular separation	*Tb*.*Sp*	*mm*	0.18	0.04	0.24	0.04	0
Trabecular thickness	*Tb*.*Th*	*mm*	0.19	0.03	0.23	0.08	0.03
Trabecular pattern factor	*Tb*.*Pf*	*1/mm*	-14.28	3.92	-10.53	3.99	0
Structure model index	*SMI*		-3.01	1.06	-2.77	1.57	0.47
Percent bone volume	*BV/TV*	*%*	65.73	6.48	63.04	10.8	0.32

All the statistical analyses were performed with R 3.2.3 [64], using the e1071 library for SVMs [65].

## Results

### Reindeer antler alveoli present a tighter mesh than red deer alveoli

#### The modern corpus

The Tb.Th variable was the only one dismissed by the normality test, due to the presence of several outliers (S1 Fig). To preserve the homogeneity of the comparisons, we chose to apply nonparametric Wilcoxon tests for all the variables. The microstructural analysis of trabecular tissue from modern antler demonstrated that reindeer alveoli are significantly smaller (Tb.Sp: 0.18 ± 0.04 mm) than red deer alveoli (Tb.Sp: 0.24 ± 0.04 mm) (Table 1). Consistent with this, their respective trabeculae were significantly more numerous (Tb.N: 3.51 ± 0.37 mm^-1^ for reindeer versus 2.83 ± 0.45 mm^-1^ for red deer). In contrast, no significant differences were observed with the variables SMI and BV/TV (Wilcoxon test, Table 1).

#### The archaeological corpus

For the archaeological corpus, only one variable, Tb.Pf, was dismissed by the normality test. We also chose to apply Wilcoxon tests for all our comparisons (S2 Fig). The alveolar mesh still appeared tighter in reindeer than in red deer. The trabecular variables were consistent with the modern group: the reindeer alveoli were smaller (Tb.Sp: 0.22 ± 0.07 mm) than those of the red deer (Tb.Sp: 0.26 ± 0.05 mm) with thinner trabeculae for the reindeer (Tb.Th: 0.20 ± 0.10 mm) than for the red deer (0.26 ± 0.09 mm). According to the Wilcoxon test, the variables Tb.N, Tb.Pf, SMI and BV/TV presented no significant differences (Table 2).

**Table 2:** Mean and standard deviation of the biometric measurements and inter-group comparisons (Wilcoxon test) for the archaeological corpus. It included 50 antler artefacts of which 23 came from red deer and 27 from reindeer (S3 Table).

			Reindeer		Red deer		Wilcoxon test
Biological parameters			*Mean*	*SD*	*Mean*	*SD*	*P-values*
Trabecular number	*Tb*.*N*	*1/mm*	2.67	0.64	2.67	0.59	0.51
Trabecular separation	*Tb*.*Sp*	*mm*	0.22	0.07	0.26	0.05	0.01
Trabecular thickness	*Tb*.*Th*	*mm*	0.20	0.10	0.26	0.09	0.02
Trabecular pattern factor	*Tb*.*Pf*	*1/mm*	-6.02	9.21	-11.25	3.62	0.10
Structure model index	*SMI*		-2.14	3.65	-3.39	1.88	0.08
Percent bone volume	*BV/TV*	*%*	53.62	25.17	65.59	11.76	0.17

### A reliable advanced statistical system to classify according to species

#### PCA confirmed the distinction between the microstructure of the two species in the modern corpus

**Fig 5:**
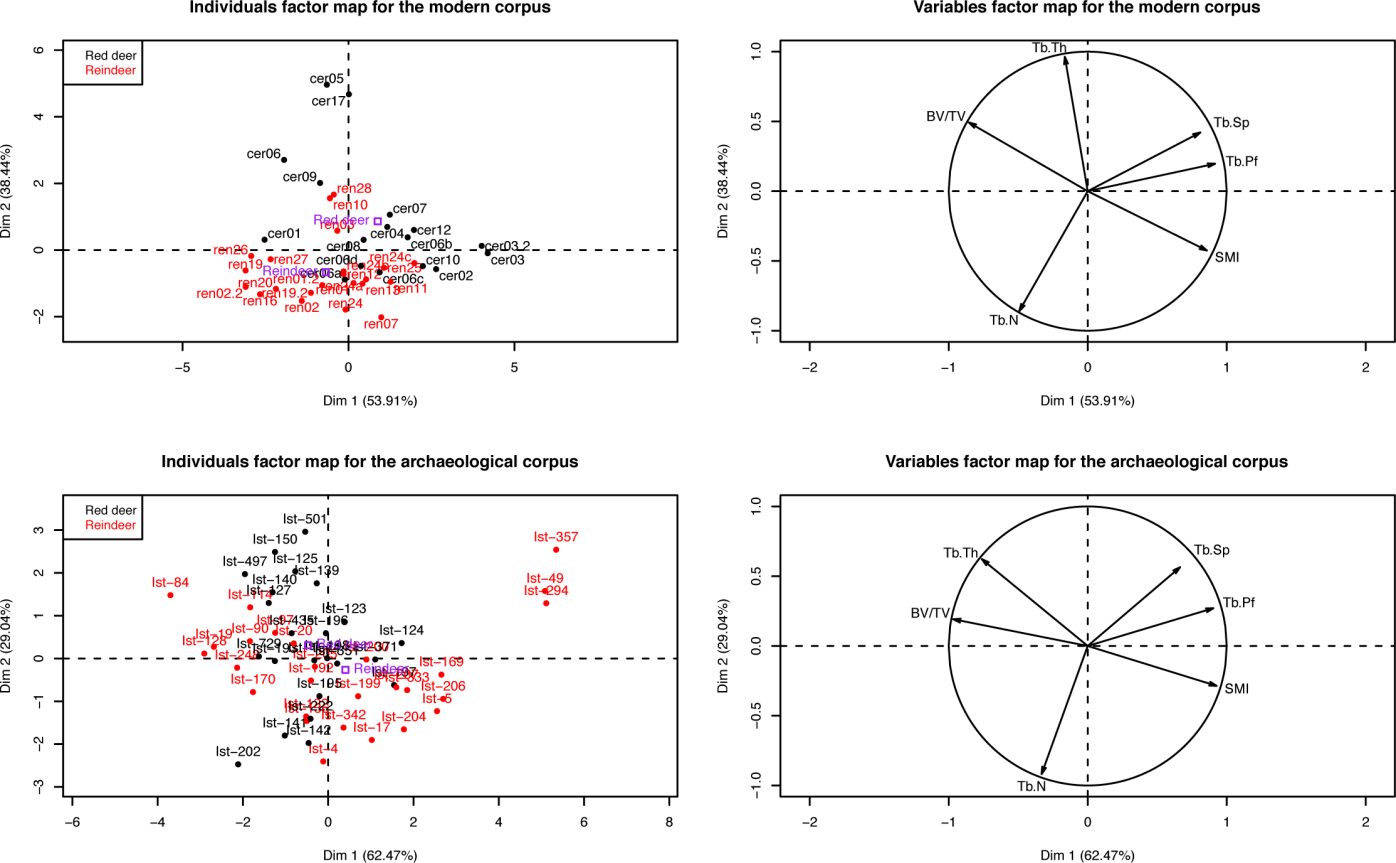
Principle Component Analysis (PCA) showing the distribution of the modern corpus and archaeological artefacts according to Tb.N, Tb.Sp, BV/TV, Tb.Pf, Tb.Th, and SMI. PCA (descriptive statistics) and the correlation circle established from the datasets described in S1 and S3 Tables. The separation on the factorial plane between both species is clearer for the modern corpus than for the archaeological one.

A Principal Component Analysis (PCA) was performed for each corpus (Fig 5). For the modern one, a clear distinction was observed between the tissue microstructures of the two species, regardless of age, sex (for reindeer), weight, geographical location, anatomical location, nature of the suture at the antler burr base (shed or slaughtered antler), and whether it involved wild or semi-domesticated individuals (Fig 5). For the archaeological corpus, the separation of the two species on the factorial plane was less clear but nonetheless visible (Fig 5). Here, the mispositioned individuals (i.e., closer to the centroid of the other species) were mostly the peripheral antler elements (ex: 357, 49, 141, and 142).

#### SVM classifiers allowed a distinction in the archaeological corpus with an accuracy of 96%

The SVM model obtained from the modern corpus leads to 7 individuals being misclassified out of the 39 tested (Table 3), with a total predictive accuracy of 82%. According to Guyon's algorithm for variable selection in SVM [66], BV/TV and SMI were the least relevant in distinguishing between the two species. In contrast, the morphometric trabecular variables produced clearer distinctions, particularly Tb.N, Tb.Th and Tb.Sp (S1 Fig).

**Table 3:** Confusion matrix from the SVM classification (predictive statistics) obtained by leave-one-out cross-validation on the modern corpus. Decision rule allowing both species to be distinguished with an accuracy of 82%.

True species \ Predicted species	Red deer	Reindeer	Total	% correct
Red deer	11	6	17	64.7%
Reindeer	1	21	22	95.4%
Total	12	27	39	82%

Nonetheless, this decision rule for modern antler could not be fully transferred to the archaeological material. Indeed, when the previous model was applied to the archaeological corpus, almost all the individuals were classified as red deer (Table 4), suggesting that significant modifications affected the biometric variables over time.

**Table 4:** Confusion matrix from the SVM classification (predictive statistics) obtained by cross-validation in which the training dataset was the modern corpus and the validation dataset was the archaeological corpus. The decision rule for modern material could not be transferred to the archaeological material (accuracy: 42%).

True species \ Predicted species	Red deer	Reindeer	Total	% correct
Red deer	18	5	23	78.2%
Reindeer	24	3	27	11.1%
Total	42	8	50	42%

The SVM model based on archaeological material alone leads to an almost perfect classification with leave-one-out cross-validation however, with a total accuracy of 96% (Table 5). Here, according to Guyon's algorithm [66], the variable that best distinguished the antler in the modern corpus (Tb.N) was less efficient for the archaeological corpus, whereas the BV/TV variable was more significant than previously observed (S2 Fig).

**Table 5:** Confusion matrix from the SVM classification (predictive statistics) obtained by leave-one-out cross-validation on the archaeological corpus. Decision rule allowing both species to be distinguished with an accuracy of 96%.

True species \ Predicted species	Red deer	Reindeer	Total	% correct
Red deer	23	0	23	100%
Reindeer	2	25	27	95.6%
Total	25	25	50	96%

## Discussion

Assuming that the bone microarchitecture analysis used in biomedical sciences could be applied to the archaeological field, we here investigated the interest and relevance of micro-CT in identifying the nature of antler artefacts. According to the related confusion matrix (Table 5), archaeological red deer antler and reindeer antler could be distinguished with an accuracy rate of 96% using a combination of bone microarchitecture criteria.

### Conservation may impact classification

According to our results, both the state of preservation of the bone tissue and the presence of sediment enclosed in the archaeological samples can impair image analysis: the fissures are treated as alveoli and the sediment appears sufficiently radiodense to be considered as trabeculae. Washing under sonication must therefore be carefully performed prior to micro-CT acquisition.

### What about identification of biomarkers within the compact tissue?

The presence of alveolar tissue is clearly essential for the method to be applied. For anthropic or taphonomic reasons, certain artefacts do not have any trabecular tissue. In this case, the identification of biomarkers directly in the compact tissue should be investigated using analytical tools that allow the microstructures (osteons) to be defined. Compact antler tissue, at the beginning of its growth, is akin to mature trabecular tissue from a histological point of view [67]. The biometric analogies observed between the mean size (0.24 mm) and number (2.83/mm^-1^ whether 11.32/4 mm^-1^) of alveoli within the mature trabecular tissue of modern red deer antler (Table 1) and those of lumens (future osteons) present within the compact tissue at different moments of its growth (size: 0.25 mm, number: 13.5/4 mm [67]) suggest that osteons could be promising taxonomic biomarkers within the compact tissue. In this regard, the recent explorative work of Blosseville [48] has produced convincing results.

### A sampling bias: the question of modules?

To explain the inability to apply the decision rule established from the modern samples to the archaeological ones (Table 4), antler modules of the two corpuses provide a useful indication. Antlers used in a Palaeolithic context mainly result from a selection of medium and large modules, particularly as regards the production of objects shaped from rod-type blanks [2]. In contrast, the constitution of the modern reference database, for reasons of age equity and probably also the inclusion of semi-domesticated individuals, led us to include a wider variety of modules and especially much higher numbers of small and very small specimens (cf., S1 Table). This finding is consistent with the results obtained in Tables 1 and 2 wherein antler alveoli from both species appear larger (and thus fewer) in archaeological specimens than in modern antlers. Overall, given that the SVM classifiers tend to maximise the distance between the nearest individuals of each group [61], the better statistical results obtained for the archaeological corpus were probably due to its more homogeneous nature.

### The potential of SVM classifiers

The SVM classification system is particularly well adapted to this archaeological case study as it considers all the biometric variables collectively, in a correlative way. Although several methods for the selection of the variables do exist [66], they did not yield better accuracies in our case. Even if the SMI and Tb.Pf variables, which correspond respectively to the architecture and connectivity of the alveolar mesh, do not appear to have a concrete biological expression here, they nonetheless help to improve the accuracy of the method from a statistical point of view. SVM classifiers maximise the potential of the method by combining biological characteristics whose individual clinical translation could sometimes appear aberrant.

### From phylogenetic to structural differences

We have shown that the structural characteristics of antler are reliable in identifying samples with a useful degree of phylogenetic precision—these two species are separated only by subfamily: Cervinae for red deer and Capreolinae for reindeer [68]. More generally, although the expression of the phenotype could be influenced by multiple endogenous (sex, age, and heredity) and exogenous (nutrition, photoperiod, accidents during development, and social relations) factors specific to each individual [69–75], the morphometric criteria identified in this study nonetheless appear to be promising biomarkers.

### Interest for archaeological studies

The internal micro-CT analysis of archaeological samples overcomes issues of human modification of the osseous raw material by scraping. Indeed, from a set of 10 products of debitage whose lower sides had been totally shaped by scraping, none were misclassified by the SVM classifiers. This is not surprising insofar as this technique transforms osseous material only at the surface level. Sample n°193 (Fig 2) confirms that this method of analysis can work for fully transformed artefacts (half-round rods) with a very small thickness of spongy tissue (2 mm). Moreover, the speed with which the samples are modelled (5 minutes per sample) allows for extensive screening applications.

Furthermore, while the anatomical location of the sample on the antler could be a disruptive variable (consistent with Léonard et al. [2007] [36], in which the porosity and size of the spongy tissue alveoli of modern red deer antler is shown to depend on the anatomical location of the sample), the archaeological corpus was not affected by this phenomenon: none of the 12 artefacts sampled that came from peripheral antler elements were misclassified by the SVM classifiers.

## Conclusion

To date, this is the first instance to our knowledge of a non-destructive method being reported, combining micro-CT analysis and SVM classifiers to distinguish archaeological antler at a species level. The biometric study performed allowed us to establish that the biological variables BV/TV, Tb.N, Tb.Sp, Tb.Th, Tb.Pf, and SMI were together relevant in distinguishing reindeer antler from red deer antler at a microscopic level. This had already been posited but not yet quantified: reindeer alveoli present a tighter mesh than red deer. The classification system, when elaborated in a predictive way (with SVM classifiers), confirmed that a distinction between the microstructures of the two tissue types could be achieved on archaeological remains (19,000–14,000 years cal BP) with an accuracy of 96%, regardless to anatomical location on the antler. This original method can allow us to build upon the macroscopic indications and physicochemical tools and identify archaeological antler artefacts according to species, thus overcoming the limitations imposed by the fragile nature, heritage value, or scientific importance of artefacts that had previously inhibited their analysis.

## Supporting Information

S1 Fig Boxplots for the biometric parameters studied (Tb.N, Tb.Sp, BV/TV, Tb.Pf, Tb.Th and SMI) for the modern samples. (TIF)

S2 Fig Boxplots for the biometrical parameters studied (Tb.N, Tb.Sp, BV/TV, Tb.Pf, Tb.Th and SMI) for the archaeological samples. (TIF)

S1 Table Deer database including the original location of the sample, age, sex, weight, semi-domesticated or wild origin, and nature of the suture at the burr base (in blue the same antler samples tested twice at two nearby locations). (TIF)

S2 Table Geographical and administrative information relative to the farms which supplied antlers (modern collection). (TIF)

S3 Table Antler industry from the Magdalenian layers (E, II, I, F1) of Isturitz cave sampled for this study. (TIF)
