# Supplementary figures and images for: A Non-Destructive Method for Distinguishing Reindeer Antler (Rangifer tarandus) from Red Deer Antler (Cervus elaphus) Using X-Ray Micro-Tomography Coupled with SVM Classifiers
Source: PLoS One. 2016 Feb 22;11(2):e0149658. doi: 10.1371/journal.pone.0149658 (PMC4762490; doi:10.1371/journal.pone.0149658)

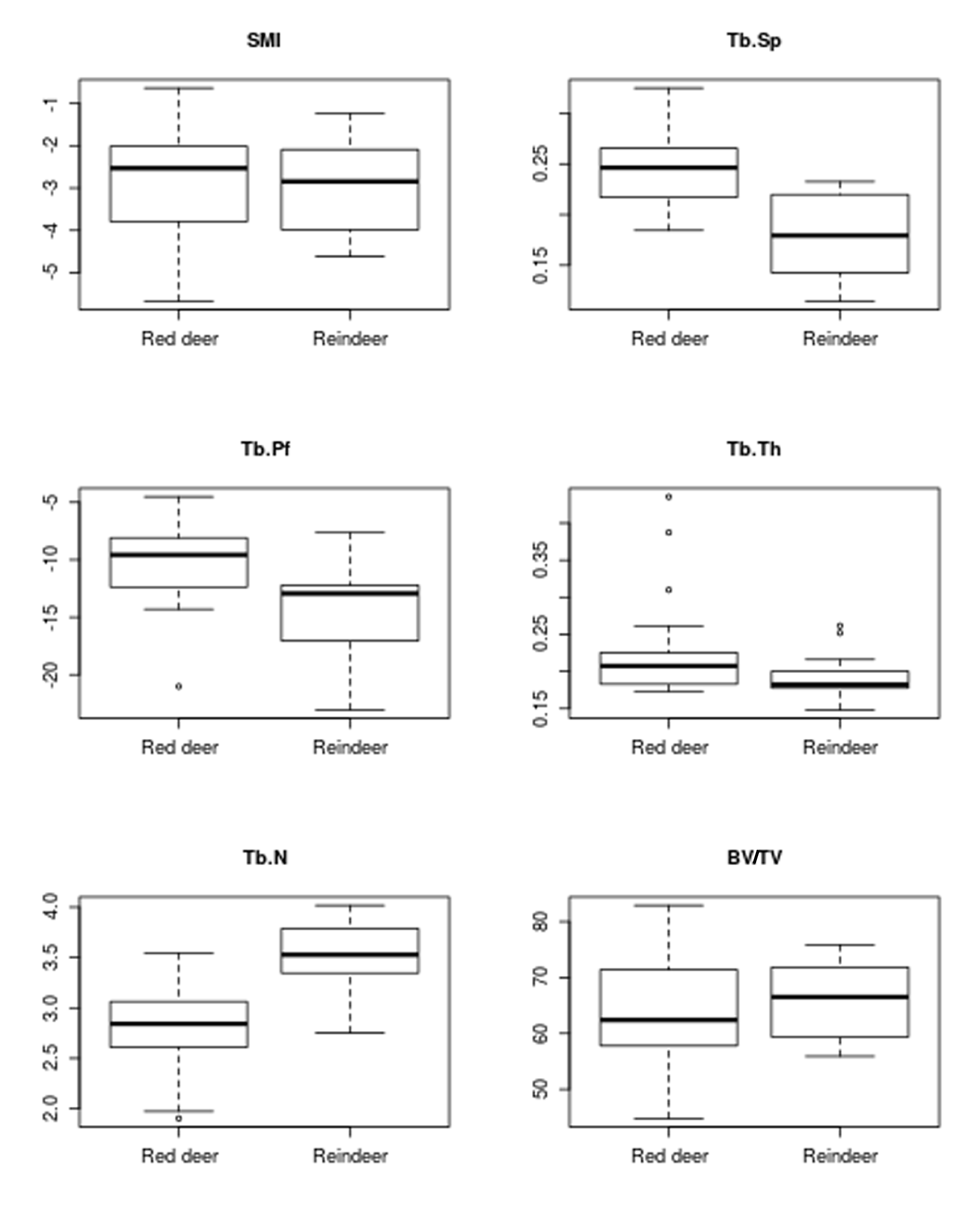

Supplement: S1 Fig — (TIF) [file pone.0149658.s001.tif]

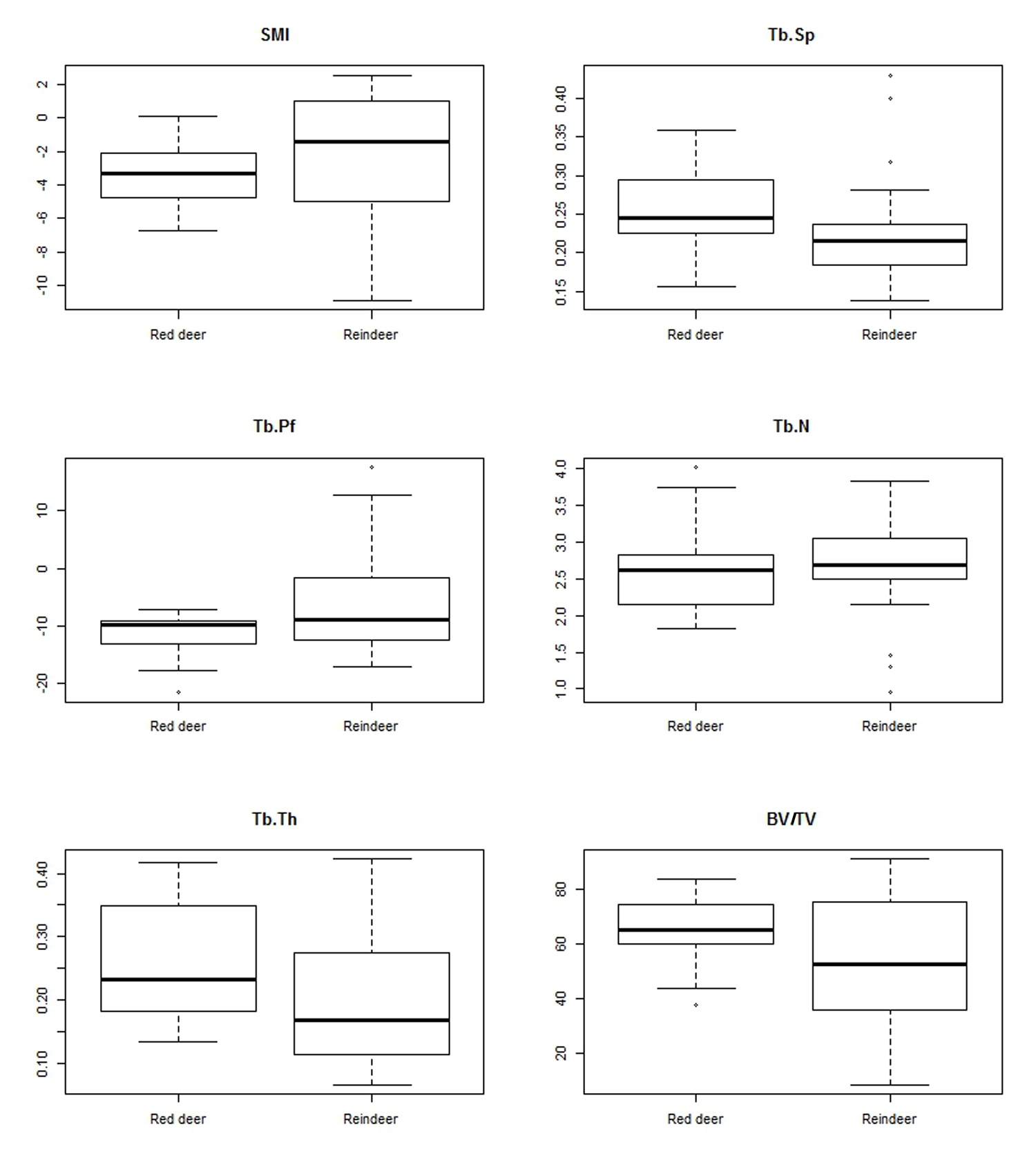

Supplement: S2 Fig — (TIF) [file pone.0149658.s002.tif]

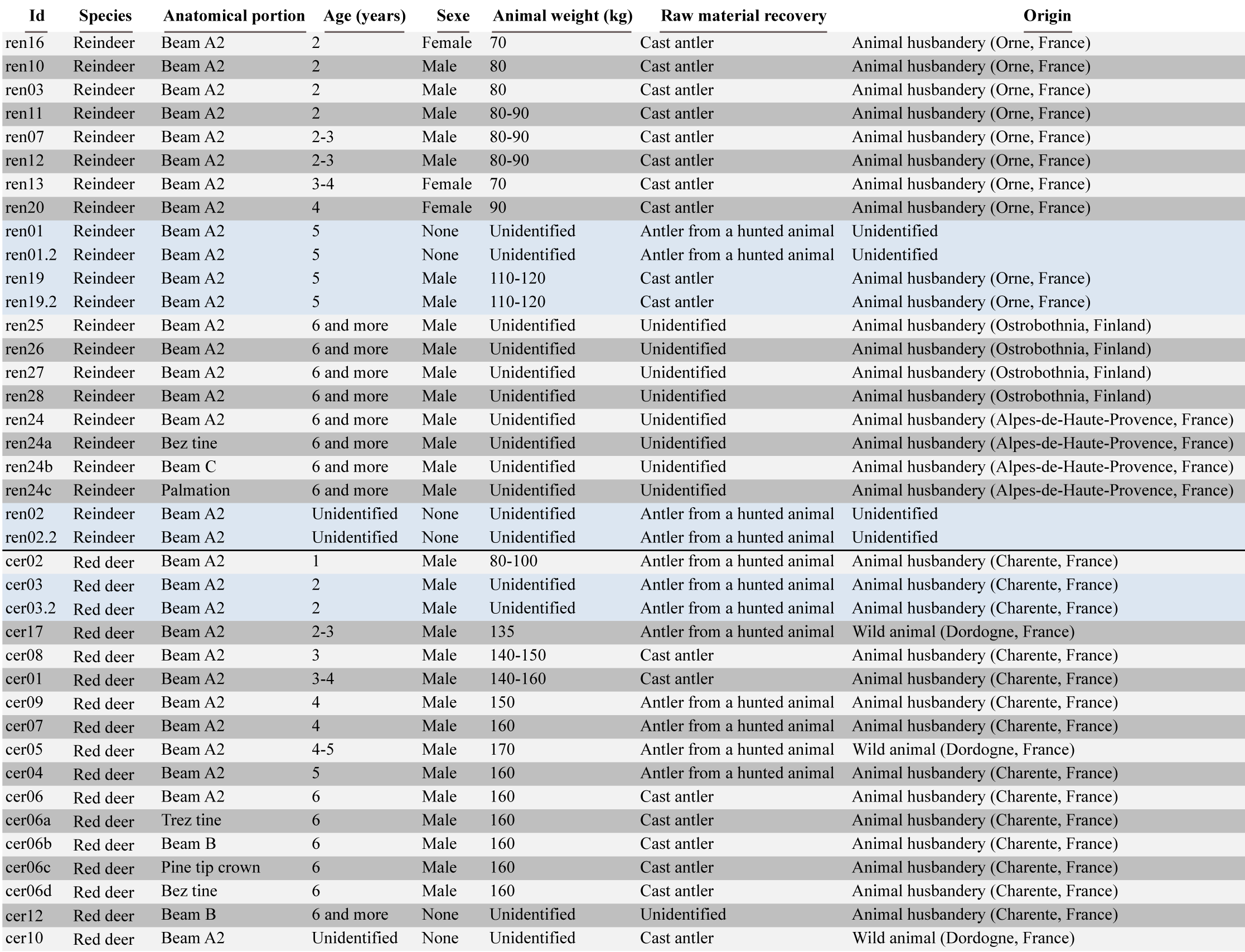

Supplement: S1 Table — (TIF) [file pone.0149658.s003.tif]

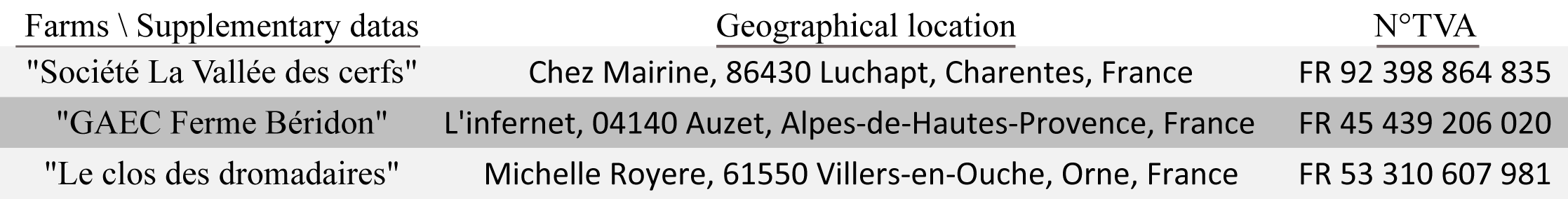

Supplement: S2 Table — (TIF) [file pone.0149658.s004.tif]

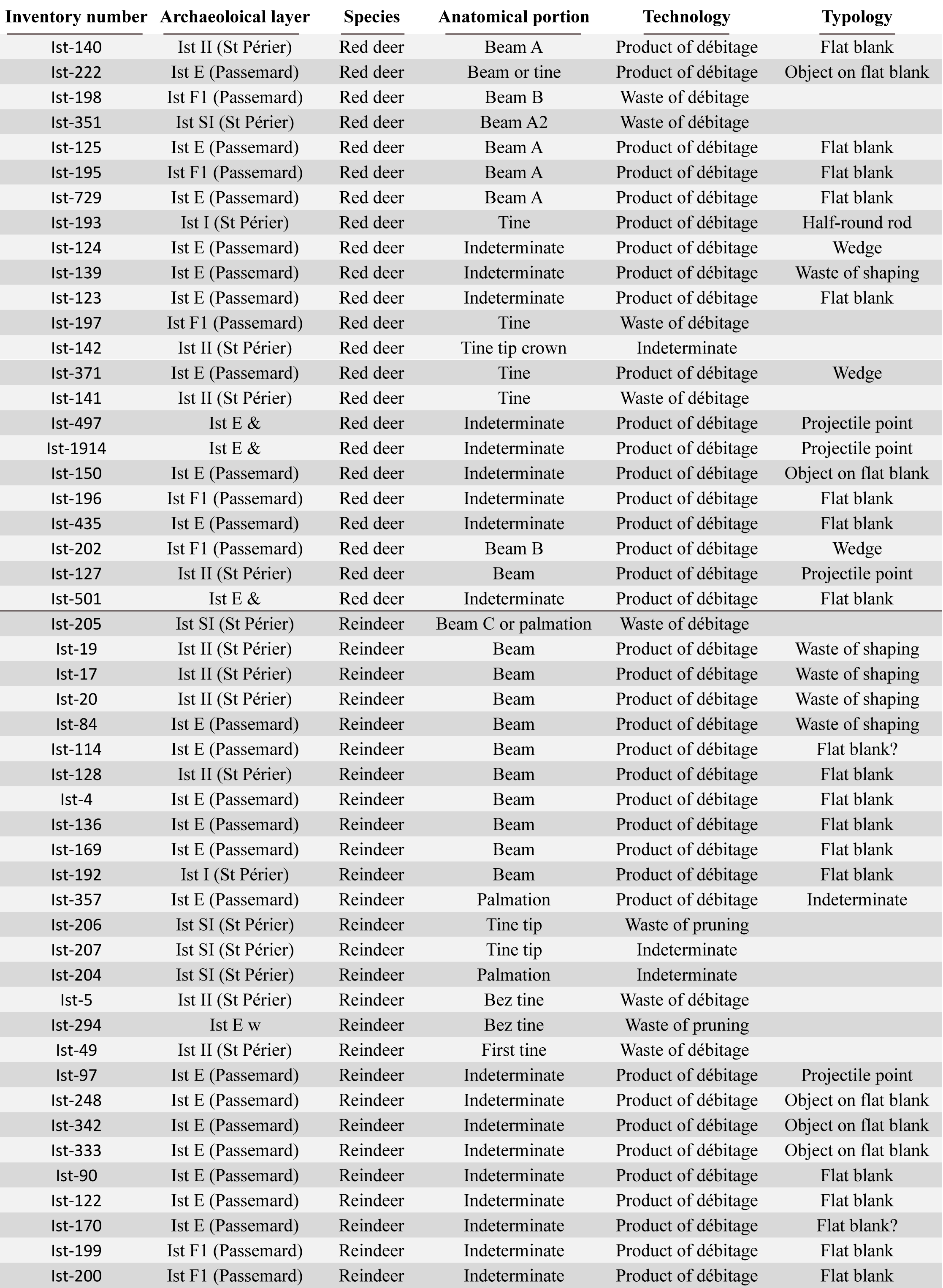

Supplement: S3 Table — (TIF) [file pone.0149658.s005.tif]
